# Identification of Laying-Related SNP Markers in Geese Using RAD Sequencing

**DOI:** 10.1371/journal.pone.0131572

**Published:** 2015-07-16

**Authors:** ShiGang Yu, WeiWei Chu, LiFan Zhang, HouMing Han, RongXue Zhao, Wei Wu, JiangNing Zhu, Michael V. Dodson, Wei Wei, HongLin Liu, Jie Chen

**Affiliations:** 1 College of Animal Science and Technology, Nanjing Agricultural University, Nanjing, People’s Republic of China; 2 Jiangsu Lihua Animal Husbandry CO., LTD, Changzhou, People’s Republic of China; 3 Department of Animal Sciences, Washington State University, Pullman, Washington, 99164, United States of America; Temasek Life Sciences Laboratory, SINGAPORE

## Abstract

Laying performance is an important economical trait of goose production. As laying performance is of low heritability, it is of significance to develop a marker-assisted selection (MAS) strategy for this trait. Definition of sequence variation related to the target trait is a prerequisite of quantitating MAS, but little is presently known about the goose genome, which greatly hinders the identification of genetic markers for the laying traits of geese. Recently developed restriction site-associated DNA (RAD) sequencing is a possible approach for discerning large-scale single nucleotide polymorphism (SNP) and reducing the complexity of a genome without having reference genomic information available. In the present study, we developed a pooled RAD sequencing strategy for detecting geese laying-related SNP. Two DNA pools were constructed, each consisting of equal amounts of genomic DNA from 10 individuals with either high estimated breeding value (HEBV) or low estimated breeding value (LEBV). A total of 139,013 SNP were obtained from 42,291,356 sequences, of which 18,771,943 were for LEBV and 23,519,413 were for HEBV cohorts. Fifty-five SNP which had different allelic frequencies in the two DNA pools were further validated by individual-based AS-PCR genotyping in the LEBV and HEBV cohorts. Ten out of 55 SNP exhibited distinct allele distributions in these two cohorts. These 10 SNP were further genotyped in a goose population of 492 geese to verify the association with egg numbers. The result showed that 8 of 10 SNP were associated with egg numbers. Additionally, liner regression analysis revealed that SNP Record-111407, 106975 and 112359 were involved in a multiplegene network affecting laying performance. We used IPCR to extend the unknown regions flanking the candidate RAD tags. The obtained sequences were subjected to BLAST to retrieve the orthologous genes in either ducks or chickens. Five novel genes were cloned for geese which harbored the candidate laying-related SNP, including *membrane associated guanylate kinase* (*MAGI-1*), *KIAA1462*, *Rho GTPase activating protein 21* (*ARHGAP21*), *acyl-CoA synthetase family member 2* (*ACSF2*), *astrotactin 2* (*ASTN2*). Collectively, our data suggests that 8 SNP and 5 genes might be promising candidate markers or targets for marker-assisted selection of egg numbers in geese.

## Introduction

Geese possess strong/variable broodiness and poor egging performances, which are impacted by many factors, such as genetics, nutrition, environment and disease. Asthe heritability of reproduction is low,it is hard to improve reproductive traits using traditional selection methods. Marker-assisted selection (MAS) is an effective way to improve such traits with low heritabilities. However, mining trait-linked sequence variationor functional genesis needed for developing MAS strategies. Single nucleotide polymorphism(SNP) is the most abundant type of genetic marker, and itshigh genetic stability makes itideal for studying the inheritance of genomic regions [1,2]. However, there is yet no genome sequence data available for geese, which largelyhinders the research of any economical traits at the molecular levelin this species.

The candidate gene approach is a common method for identifying genetic markers linked to important economical traits. Chen et al (2012) found more than 30 SNPs in *Prolactin* (*PRL*) intron 2, and 5 SNPs in *Prolactin Receptor* (*PRLR*) exon 10 in *Wanjiang* white geese. These polymorphisms were significantly related to the egg productivity [3]. Zhao et al (2011) found two SNPs respectively on *Gonadotropin-releasing Hormone* (*GnRH*) and *PRL* were associated with reproduction traits in *Wulong* geese [4].Zhang et al (2013) demonstrated the gene expression of *Luteinizing Hormone* (*LH*), *PRL* and their receptors at different stages in *Zi* geese [5], and Ding et al (2006) identified many differentially expressed genes in livers of laying geese compared with prelaying geese using suppression subtractive hybridization (SSH). These genes included *Vitellogenin I*, *apoVLDL-II*, *ethanolamine kinase*, *G-protein gamma-5 subunit*, and *leucyl-tRNA synthase*[6]. Recently, Guo et al (2011) used a similar approach to find several differentially expressed genes between the laying and broodiness stages, including *PRLR*, *estrogen receptor 1* and *anti-mullerian hormone receptor II*[7].

Next-generation high-throughput DNA sequencing techniques have accelerated theresearch speed of animal genomic research. This techniques has been widely used in whole-genome sequencing, target resequencing, and transcriptome sequencing[8]. Most recently, Xu et al (2013) identified 572 differentially expressed genes with 294 up-regulated and 278 down-regulated genes in the ovarian tissue library of laying geese and broodiness geese by *de novo* transcriptome assembly using short-read sequencing technology (Illumina) [9]. Unfortunately, the resultant transcriptome provided only limited restriction site information from coding regions, where nucleotide diversity is much lower compared to non-coding regions.

Restriction-site associated DNA (RAD) sequencing, a newly developed method for rapid and large-scale SNP discovery, can effectively reduce the complexity of the genome[10]. It has becomean economical and efficient method for SNP discovery and genotyping [11,12]. It allows smaller research groups, or groups studying organisms that do not yet possess a reference genome, to conduct “genome wide studies”[13]. The RAD sequencing approach has been successfully applied in a number of organisms, including guppy [14], salmon [15], eurasian beaver [16], cutthroat and rainbow trout [17], Sturgeon [18], and rapeseed [10].

In this study, we applied pool-based RAD sequencing to discover novel SNP across the goose genome. Candidate SNP for laying performance were selected by comparing allelic frequencies between the two DNA pools with lowest estimated breeding value (LEBV) and highest estimated breeding value (HEBV). Using an allele-specific PCR (AS-PCR) assay for individual-based genotyping, the candidate SNP-traitassociation pattern was first confirmed in LEBV and HEBV cohorts, and further verified in the population of 492 geese. Novel genes harboring laying-related SNP were cloned for geese.

## Materials and Methods

### Ethics Statement

All experiments were reviewed and approved by Nanjing Agricultural University Animal Care and Use Committee and performed in accordance with the Regulations for the Administration of Affairs Concerning Experimental Animals (China, 1988). All efforts were made to minimize any discomfort during blood collection.

### Animals and Sample Preparation

A total of 492 female *Yangzhou* geese from the breeding farm of Jiangsu Lihua Animal Husbandry CO., LTD were employed in this study. During the experiments, geese were fed ad libitum with rice grain supplemented with green grass or water plants whenever possible. The feed was offered during daytime when the geese were released to an open area outside the house. The geese were exposed to natural lighting and temperature throughout this study. The laying geese were kept in separate cages in order to record the total number of eggs during the whole egg-laying period. Blood samples were collected from wing vein using sodium heparin containing vacutainers.

### Laying Performance and Grouping

The total egg number of all individuals was recorded daily throughout the egg-laying periodof 34 weeks. The average egg numbers of the experimental population aresummarized in Table 1. Individual estimated breeding values (EBV) of egg numberwas calculated using the information of full sib and half sib. Ten individuals of lowest or highest EBV were selected from the total 492geese and designated LEBV and HEBV groups, respectively.

**Table 1:** Egg numbers of the experimental population in 34-weekegg-laying period.

Population	Number of geese	EBVs	Average egg number
All geese	492	74.08±8.01	73.95±19.73
LEBV	10	58.87±3.97	34.9±12.73
HEBV	10	87.42±4.18	105.6±3.98

### RAD Library Preparation and Sequencing

Genomic DNA was extracted from blood using the whole blood DNA kit (Omega Bio-Tek, Doraville, USA) following the manufacturer’s instructions. DNA concentration was assessed for each individual sample using the Thermo Scientific NANODROP2000 spectrophotometer(Thermo Fisher Scientific Inc. USA). All DNA samples were adjusted to a final concentration of 100ng/ul. Both A260/280 and A260/230 ratios were in the standard range. Two DNA pools for LEBV and HEBV were prepared by mixing equal amount of genomic DNA from each 10 individuals. The restriction enzyme EcoRI was used to digest the genomic DNA. A total of 2 multiplexed sequencing libraries were constructed, in which each DNA sample was assigned a unique nucleotide multiplex identifier (MID) for bar-coding. Single-end (101-bp) sequencing was performed using Illumina HiSeq2000.

### Sequence Analysis and Laying-Related Mutations Detection

Raw sequence reads were trimmed to 90 nucleotides from the 3’ end, which ensured more than 97.5% of the nucleotides have a quality value above Q30 (equals 0.1% sequencing error). The trimmed reads were clustered into read tags (hereafter RAD-tags) by sequence similarity using USTACKS [19] to produce unique candidate alleles for each RAD locus. A maximum base-pair mismatch of two was allowed in this step for the natural populations. RAD-tags were then collapsed into clusters using USTACKS under default parameters for SNP calling. For each SNP, the differences of allele frequencies were compared between LEBV and HEBV pools. Those SNP with significantly different allelic distributions between the two pools were chose as candidate loci for the further verification in the population.

### Verification of Laying-Related Mutations in Goose Population

A total of 55 SNP were selected for further individual-based genotyping in the LEBV and HEBV cohorts. The SNP having different allelic distribution between LEBV and HEBV cohorts were verified in the population of 492 geese. AS-PCR was used for genotyping in the population. In order to improve the specificity of PCR amplification and reliable discrimination between the alleles, an additional mismatch base pair was introduced at the third base from 3’ end. The primers of AS-PCR were designed according with the methods of Liu[20]and Hayashi [21] by Primer Premier 5 software (PREMIER Biosoft, Palo Alto, CA, USA).

The primers and PCR production length are showed in S1 Table. Genotyping with two specific primers was performed duplicated in 20μL reactions containing approximately 50 ng template DNA, 5 μl 2X PCR Taq enzyme (abm, Canada), 1μl of specific and common primer (10 μmol) (BGI, ShenZhen, China). Amplification conditions were as following: predenaturation at 94°C for 3 min, 32 cycles of amplification (94°C for 30s, 45°C-72°C for 30s and 72°C for 30s) and a final extension at 72°C for 5 min. PCR products were separated on 3.0% agarose gel by electrophoresis.

### Cloning Novel Genes Based on Egg Laying-Related SNP

Functional genes harboring verified laying-related SNP were further cloned for geese using inverse PCR (IPCR) coupled with comparative sequencing. IPCR is a method for amplifying unknown sequences (adjoining known sequences) by primers designed on the known sequence in opposite orientation using self-ligated circular DNAs as PCR templates. All primers were designed base on the sequence of RAD tags. The primers used in this work are listed in S2 Table. Five microgram of genomic DNA was digested in a 200ul total volume using Kpn I, Hind III, Sac I and Noc I (All the enzymes from NEB, Beijing, China) at 37°C for 6 hr to achieve complete digestion. The digested sample was then treated with an equal volume of Phenol: chloroform: isoamylalcohol (25:24:1) mixture, the aqueous phase was removed, and the DNA was precipitated with ethanol and collected by centrifugation. The digested DNA was self-ligated at a concentration of 0.5–1.0 ug/ml in the presence of 1600U/ml T4 DNA ligase (NEB, Beijing, China) overnight at 16°C. The ligation mixture was extracted by Phenol: chloroform: isoamylalcohol (25:24:1), precipitated with ethanol, and resuspended in sterile distilled water to a concentration of 50 ng/ul.

Nest PCR was applied for amplifing unknown sequence flanking the RAD-tag. Nest PCR was performed in a volume of 50 ml with 50 ng prepared DNA, 2μl of each of primer (10 μmol) (BGI, ShenZhen, China) and 25ul LA Taq enzyme (Takara, DaLian, China). Amplification conditions were as followed: pre-denaturation at 98°C for 30 s, 32 cycles of amplification (98°C for 10s, 45°C-72°C for 30s and 72°C for 4min) and a final extension at 72°C for 7 min. After the first round PCR, diluted the PCR production 1:100 with double distilled water. 1ul of the diluted solution was then used as the template for the second round amplification. The primer W (outer primer pairs) and N (inner primer pairs)were used for thefirst and second PCR amplification, respectively. PCR reaction mixtures were analyzed on a 1.5% Tris/Boric acid/EDTA (TBE) agarose gel. PCR bands were excised under UV light and purified using the gel extraction kit (Omega Bio-Tek, Doraville, USA) as recommended by the supplier. The purified DNA fragments were directly ligated into a phagemid TA vector (Peasy-T3 plasmid) using the TA cloning kit (TransGen Biotech, BeiJing, China) according to the manufacturer’s protocol, and then transformed to the competent cells (TransGen Biotech). Transformants were plated on LB agar containing 50 mg/ml ampicillin. Colonies were selected and sampled, suspended in 1 ml of LB medium in the 2.0 Eppendorf tube and grown at 37°C for 16 hr. The target DNA was sequenced (GENEWIZ, Suzhou, China). Multiple sequence alignments were performed using DNAman software package(Version 8.0; Lynnon Bio-Soft, Quebec, Canada). Database searches were performed using BlastX (http://www.ncbi.nlm.nih.gov/BLAST).

### Statistics and Data Analysis

The chi square test of independence was used to test the difference of allelic frequencies of RAD-tags between LEBV and HEBV DNA pools. For the discovery of laying-related SNP, the Bonferroni correction was used toestimate the significance thresholdat the 5% overall Type I error rate[22], *α*_*B*on_ is given by

$$\alpha_{Bon}\;=\;\frac{\alpha }{n_{informative}}$$

Where *α*_*B*on_ is the Bonferroni-adjusted P value, *α* is the uncorrected P value, *n*_*informative*_ is the number of SNP.

Fisher’s exact test was executed in the statistical language R version 2.11.1[23]to compare the allelic frequencies between LEBV and HEBV cohorts.

The laying estimate breeding value (EBV) of individual was calculated as:

A^=∑biPi=b′P

Where A^ is the EBV. ***b***_i_ is the phenotypic information of the i ^th^ relatives, including phenotypic performance of individual, full sibs and half sibs. ***b***_i_ is the partial regression coefficient of ***P***_i_. **b′** is the vector of partial regression coefficient. **P** is the vector of phenotypic value.

Genotype frequencies, allelic frequencies, gene diversity, heterozygosity, polymorphism information content (PIC) and chi-square tests of goodness-of-fit for Hardy-Weinberg equilibrium law were calculated using PowerMarker V3.25[24]. All data were expressed as the mean ± SD.

One-Way ANOVA (SPSS for Windows, version 20.0; IBM-SPSS, Chicago, IL) was used to compare average egg numbers of the different genotypes. The means were assessed for significance by Duncan’s multiple range tests (SPSS for Windows, version 20.0). All single SNP-trait associations that reached a significance level of *p<0*.*05* were included in further multiple-marker analysis. Multiple-marker associations were analyzed along with two quantitative trait modes (additive mode: P_Aa_ ≈ (P_AA_ + P_aa_)/2) and dominant mode: P_Aa_ ≈ either P_AA_ or P_aa_) by the linear regression procedure (SPSS for Windows, version 20.0)[25].

## Results

### RAD Sequencing

RAD sequencing generated 3.8 Gb of data containing more than 42.29 million single-end reads, with each read being 90 bp in length (Table 2). The RAD-tags were aligned within-group and inter-group, with the number of mismatch was 1. The number of RAD tags per group is 884,827 and 942,117 for LEBV and HEBV respectively. The sequencing depth per group is 17.33× and 20.47× respectively, with average sequencing depth of 18.9×. After the filtering steps, a total of 139,013 SNP were detected. Only the SNP distributed from position 6 to 90 were chose for further analysis, since the polymorphisms beyond this region were more subjected to common sequencing errors. Of all SNP, 338 were triallelic. The remaining 138,675 SNP were biallelic and consisted of 52.97% transitions and 47.03% transversions, providing a transition/transversion (ts/tv) ratio of 1.10. The number of A/G substitution (38,549)almost equaled the number of C/T substitution (34,226) in the transitions class, while G/T (31,622) transversions exceeded A/C (12,384), A/T (13,078) and C/G (8,816) transversions.

**Table 2:** Restriction-site associated DNA (RAD) sequencing statistics of EcoRI library from two DNA pools.

Sample	Clean Data	Clean Reads Number	RAD-tag number	Average Depth
LEBV	1,727.02Mb	18,771,943	884,827	17.33
HEBV	2,163.79Mb	23,519,413	942,117	20.47

### Discovery of Laying-Related SNPs

The differences of allelic frequencies betweenLEBVandHEBV pools were analyzed by chi-square tests for all 138,675 SNP of RAD sequencing. After Bonferroni adjustment, 467 SNP were significant (*p*<3.69×10^−7^). Individual-based genotyping were performed for all LEBV and HEBV geese by allele specific-PCR. Totally, 55 SNP could be stably genotyped by this method (S1 Table). The results of further individual-based genotyping showed that 10 out of 55 SNP had significant (*p<*0.00024–4.19×10^−8^) different allelic frequencies in the LEBV and HEBV cohorts (Table 3).

**Table 3:** Allelic frequencies distribution between LEBV and HEBV cohorts.

SNP	primer	genotype	HEBV (Allele frequencies)	LEBV (Allele frequencies)	P value
Record-106975	AATTCTTGCCTAAATAAC(A/G)	G/A	0.10/0.90	1.0/0	4.19×10^−8^
	ACACATTGATGCTGCAAATT				
Record-134172	GCTGACAGCTCATTTGAT(A/T)	A/T	1.0/0	0.30/0.70	3.34×10^−6^
	CAGGATCACGTCCTCAAC				
Record-112359	GAAGCGCCTGGTGGCCTCACC(T/G)	T/G	0/1.0	0.80/0.20	7.71×10^−7^
	TACCTGCCTGAGAGATGGAGTGTTGG				
Record-106582	TTCAGGGAACTCAAACTATA(C/A)	C/A	0.20/0.80	0.90 /0.10	1.66×10^−5^
	CTCACGTGGCCTCTACAA				
Record-111407	TTTTGGCCGTGGTTTCC(T/A)	T/A	0.40/0.60	1.0/0	4.51×10^−5^
	ATCAGACCCGTGGTGGAAC				
Record-135849	CTGTGTTGATATTTTCTGATAGTA	T/C	0.70/0.30	0.10/0.90	2.44×10^−4^
	TGTTTAGCAGGAGCACAA(T/C)				
Record-88247	AGAGAATTTAGTCATATTTGGGT	G/A	0.21/0.79	0.85/0.15	2.20×10^−5^
	TAATTTGGACTCAATCAAAA(C/T)				
Record-135057	CAGGGGGATTCTAGTAGTTTCCT	G/A	0.14/0.86	0.80/0.20	6.34×10^−6^
	TCACTGCTGCAGACACGCA(T/C)				
Record-130652	AGTTACATTTAAAAGTGTCTGGTC	G/A	0.75/0.25	0.05/0.95	1.02×10^−6^
	CGATCAAAACAAATGGTAGAC(C/T)				
Record-130775	CAGGGGGATTCTAGTAGTTTCCT	A/G	0.96/0.04	0.10/0.90	4.63×10^−10^
	TCACTGCTGCAGACACGCA(T/C)				

### Verification of Laying-Related SNP in the Experimental Goose Population

**Fig 1:**
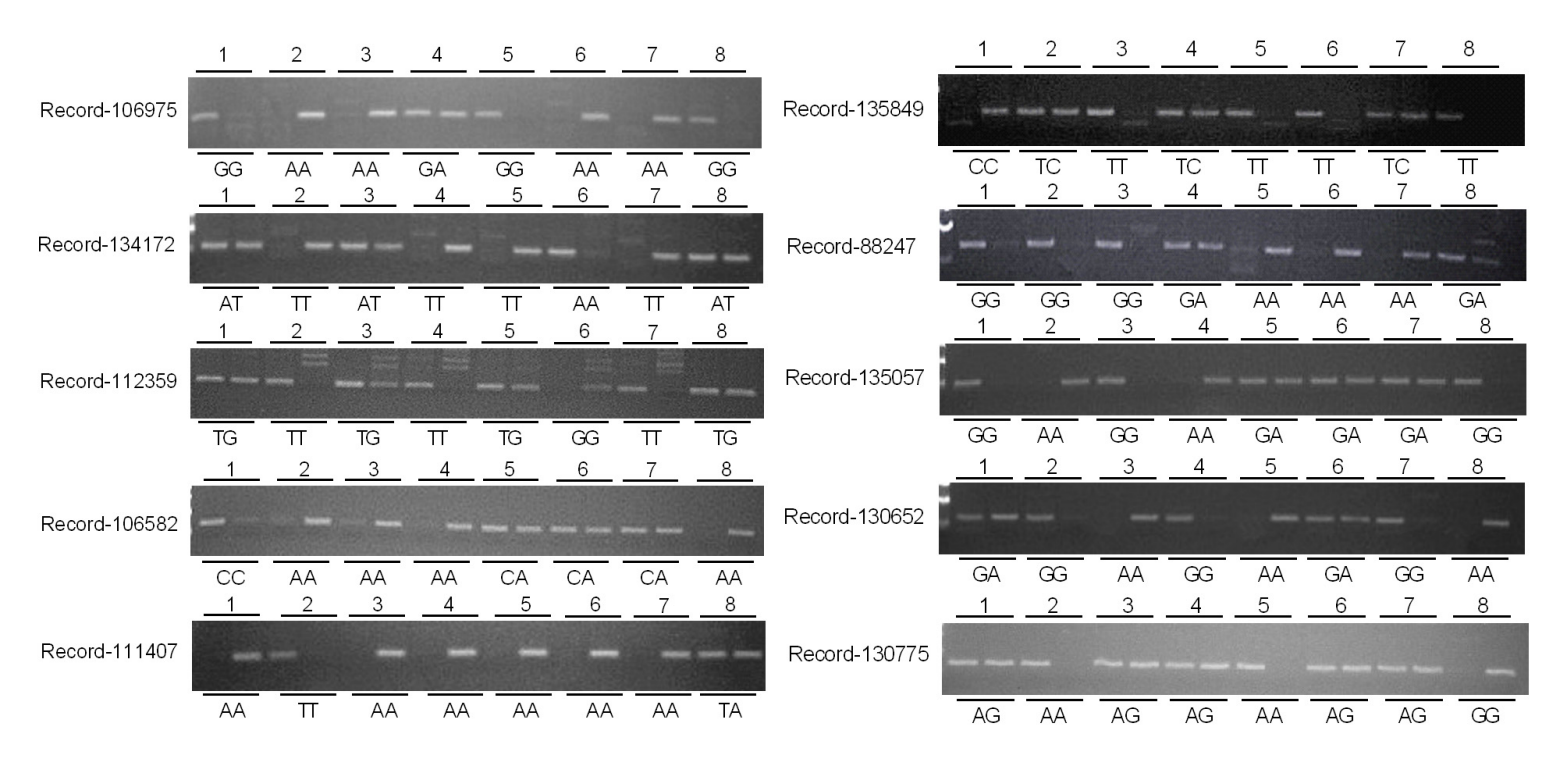
Genotyping of the 10 SNP by allele specific PCR in geese. Electropherosis of AS-PCR revealed different genotypes of 10 SNPs. Eight individuals were randomly selected to show three genotypes of each SNP.

The 10 SNP were subjected to genotyping in the population of 492 geese by AS-PCR (Fig 1). Genetic diversity was analyzed by software Powermarker V3.25 for each SNP. As shown in Table 4, the gene diversity (He), heterozygosity (H0), and polymorphism information content (PIC) of the 10 SNP ranged 0.4394–0.4991, 0.0830–0.5233 and 0.3161–0.3746 respectively. The SNP Record-135849 had the highest gene diversity, heterozygosity and PIC. PIC commonly used as a measure of polymorphism in genetics for a molecular marker. In the present study, the PIC values of 10 SNPs range from 0.25–0.5, indicates that these SNPs display intermediate levels of polymorphism.Seven SNP showed significant deviations from Hardy-Weinberg equilibrium (HWE) (*p*<0.05), while the other three SNP, including Record-135849, Record-88247 and Record-135057, were in HWE (*p*>0.05).

**Table 4:** SNP Identification and Genotyping by allele specific PCR: genotype and allele frequencies, polymorphism information content, and chi-square tests of goodness-of-fit for Hardy-Weinberg equilibrium in the experimental population.

SNP	Sample size	Genotype frequencies (%)			Allele frequencies(%)		He	H0	P valve (χ2,HWE)	PIC
Record-106975	449	GG25.4	GA30.1	AA44.5	G40.4	A59.6	0.4817	0.3007	0.0000*	0.3657
Record-134172	332	AA46.7	AT33.7	TT19.6	A63.6	T36.4	0.4633	0.3373	0.0000*	0.3560
Record-112359	330	TT35.4	TG39.7	GG24.9	T55.3	G44.7	0.4944	0.3970	0.0001*	0.3722
Record-106582	472	CC32.6	CA42.6	AA24.8	C53.9	A46.1	0.4969	0.4258	0.0018*	0.3735
Record-111407	457	TT43.5	TA38.7	AA17.8	T62.9	A37.1	0.4669	0.3868	0.0002*	0.3579
Record-135849	193	TT21.8	TC52.3	CC25.9	T47.9	C52.1	0.4991	0.5233	0.5544	0.3746
Record-88247	260	GG53.9	GA38.4	AA7.70	G73.1	A26.9	0.3935	0.3846	0.6344	0.3161
Record-135057	341	GG47.5	GA41.6	AA10.9	G68.3	A31.7	0.4328	0.4164	0.5060	0.3391
Record-130652	342	GG12.6	GA32.2	AA55.2	G28.7	A71.4	0.4089	0.3216	0.0002*	0.3253
Record-130775	446	AA53.4	AG8.30	GG38.3	A57.5	G42.5	0.4887	0.0830	0.0000*	0.3693

Note: PIC means polymorphism information content.

^*^means significance at the *p<* 0.01 level.

As shown in Table 5, the GG and GA genotypes of Record-106975 had significantly higher egg productionthan those with AA genotype(*p<*0.01). No significant difference in egg production was observed between the GG and GA genotypes (*p*>0.05).

**Table 5:** Least squares means and standard deviations of the association analysis between different genotypes on egg number.

SNP	Genotype	Number	Egg production	P value
Record-106975	GG	115	77.83±24.28^B^	2.0×10^−4^
	GA	133	77.64±23.20^B^	
	AA	201	67.24±20.76^A^	
Record-134172	AA	155	74.48±24.99^B^	0.001
	AT	112	64.11±21.29^A^	
	TT	65	69.78±18.22^A^ ^B^	
Record-112359	TT	117	76.41±22.31^B^	4×10^−4^
	TG	131	69.17±24.46^A^ ^B^	
	GG	82	62.50±19.07^A^	
Record-106582	AA	154	78.66±22.18^B^	4×10^−4^
	CA	201	75.91±21.74^B^	
	CC	117	64.41±22.19^A^	
Record-111407	AA	82	81.05±23.02^B^	4×10^−6^
	TA	177	75.99±21.45^B^	
	TT	198	66.64±222.14^A^	
Record-135849	TT	42	69.46±32.02	0.054
	TC	101	72.20±21.25	
	CC	50	61.46±25.83	
Record-88247	AA	20	82.75±26.54^A^ ^a^	5×10^−5^
	GA	100	70.05±22.73^b^	
	GG	140	64.36±23.22^B^	
Record-135057	GG	162	65.76±21.67^A^	9×10^−6^
	GA	142	73.61±24.33^B^	
	AA	37	78.70±28.13^B^	
Record-130652	GG	43	74.19±23.85	0.059
	GA	110	73.81±26.64	
	AA	189	67.73±22.08	
Record-130775	AA	238	77.26±23.48^A^	2×10^−6^
	AG	37	74.86±18.83^A^	
	GG	171	65.64±21.30^B^	

Note: Multiple comparisons were performed using the Duncan multiple-range test

^a, b^ means with different superscripts in the same column are different with *p<*0.05

^A, B^ means with different superscripts in the same column are different with *p<*0.01.

SNP Record-134172 geese with AA genotype showed higher egg production in comparison to the geese with AT genotype (*p<*0.01), but TT genotype showed no significant difference with the AA and AT genotypes (*p*>0.05).

Record-112359, the TT genotype had significantly higher egg production than those with GG genotype (*p<*0.01). The TG genotype showed no significant difference on egg production compared with the TT and GG genotype (*p*>0.05)

Record-106582, the AA and CA genotype had significantly (*p<*0.01) higher egg production than those with GG genotype, but there was no significant difference between the CA and AA genotype (*p*>0.05).

Record-111407, geese with the AA and TA genotype had significantly higher egg production than those with the TT genotype (*p*<0.01). No significant difference in egg production was observed in the AA and TA genotypes (*p*>0.05).

For Record-88247, the AA genotype had significantly higheregg production than those with GG genotype(*p<*0.01). The AA genotype had significantly higher egg production than those with GA genotype (*p<*0.05).

For Record135057, the AA and GA genotypes had significantly higher egg production than those with GG genotype (*p<*0.01).

For Record-130775, the AA and AG genotypes had higheregg production than those with GG genotype (*p<*0.01).

For Record-135849 and Record-130652, no significant association between the genotypes and egg production was found (*p*> 0.05). These results indicated that these 8 SNP (Record-106975, Record-134172, Record-112359, Record-106582, Record-111407, Record-88247, Record-135057, Record-130652 and Record-130775) were significantly associated with egg production trait (*p*<0.01).These newly identified SNP were deposited in NCBI dbSNPdatabase with the accession numbers 1714766361, 1714766362, 1714766363, 1714766364, 1714766365, 1714766367, 1714766368 and 1714766370, respectively.

### Regression Analysis of Multiple Markers on Laying Performance

**Fig 2:**
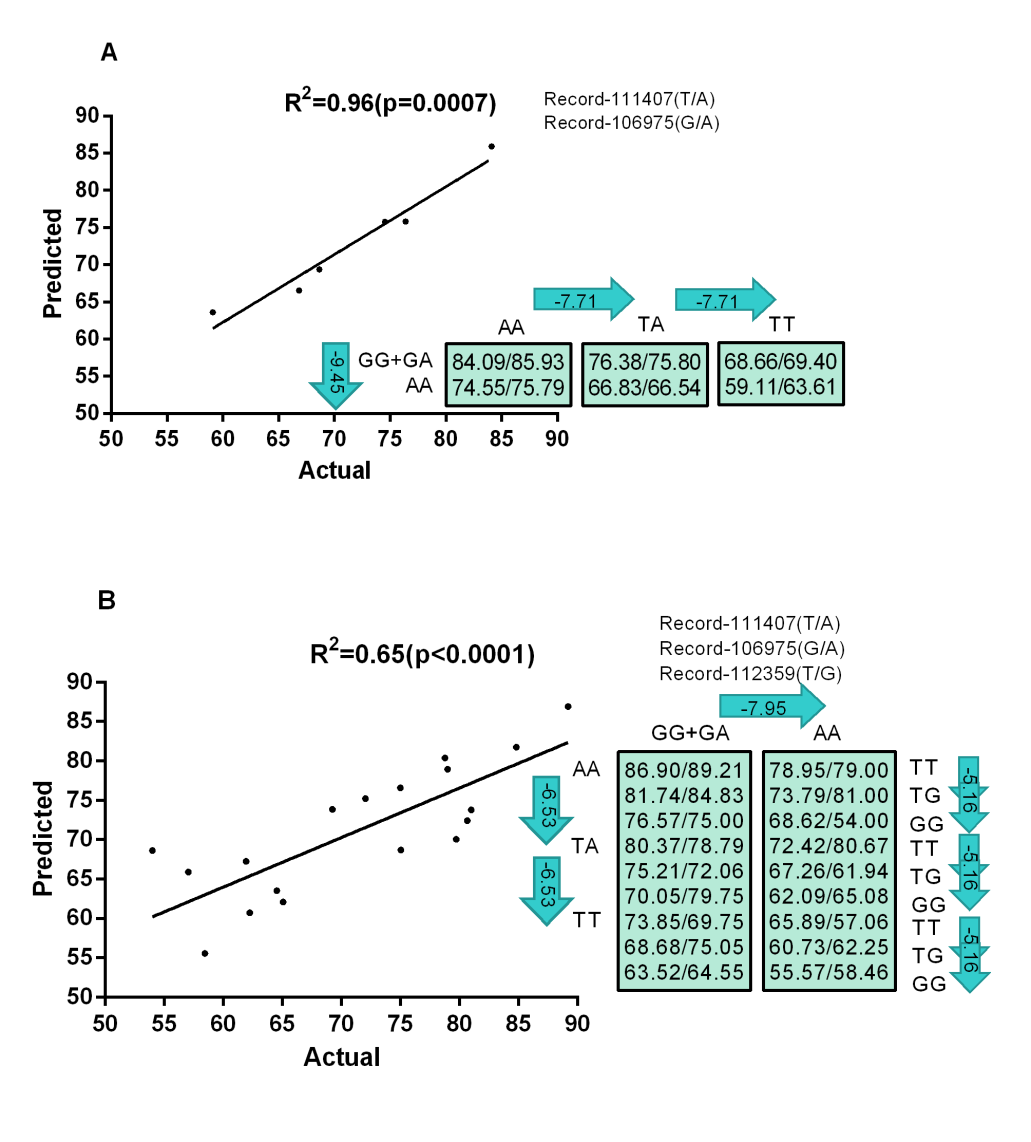
Genetic networks with multiple makers established by the linear regression analysis for egg number in geese. The numbers in arrows represent substitution effects of one type of genotypes or allele for another one. Each combined genotype(s) between different makers has two means of performance: predicted (left in the rectangle) and actual (right in the rectangle) values. A) Genetic networks with two markers established by the linear regression analysis for egg number in geese. B) Genetic networks with three markers established by the linear regression analysis for egg number in geese.

In the single-marker associations, we identified 8 SNP with significant effects on egg numbers in geese. Linear regression model analysis was used to evaluate multiple significant markers effect on goose laying performance. The 8 SNP were involved in the analysis to determine gene combinations or networks for the trait (Fig 2). Two networks were established, one of which included two markers, and another consisted of three markers. Record-111407 and Record-106975 were included in the two-marker network (Fig 2A). The predicted value (left in the rectangle) showed high correlation with corresponding actual values (right in the rectangle) (r = 0.98, r = 0.81). Record-111407 and Record-106975 demonstrated additive and dominant effect on laying performance, respectively. For Record-106975, the substitution of GG/GA with TT genotype will lead to decreasing of average egg numbers by 9.45. For Record-111407, transversion of A to T will result in descend of egg numbers by 7.71. The three-marker network introduced an additional marker Record-112359, which exhibited additive effect on laying performance (Fig 2B). The substitution of T to G decreased egg numbers by 5.16.

### Identification of Novel Genes Harboring Laying-Related SNP

Based on the above 8 candidate SNP, we tried to identified related functional genes in goose. Firstly, BLAST searches against the NCBI public database using the candidate RAD tags were performed to retrieve orthologous sequences. However, no significant matched sequence was obtained since the 90-bp RAD tags were too short for effective alignment. Therefore, we used IPCR to extend the unknown regions flanking the candidate RAD tags. The extended sequences were used for further BLAST. As there is no reference genomic information available for geese yet, we mainly used the retrieved duck or chicken sequences for goose gene annotation.

As shown in Table 6 and S2 Table, we cloned 2,488 bp length of flanking sequence based on Record-106975. The DNA sequence exhibits 89% and 65% identities with duck and chicken sequences in the whole-genome shotgun contigs database of ducks (taxid: 8835) and chicken (taxid: 9031), respectively. The gene of *membrane associated guanylate kinase*(*MAGI-1*) was obtained base on the sequence of Record-106975 by the tool of NCBI Map Viewer.

**Table 6:** Nucleotide BLAST and gene cloning.

SNP	DUCK			CHICKEN				
	Accession number	Query cover (%)	Identity (%)	Accession number	Query cover (%)	Identity (%)	Chromosome	Gene
Record-106975	ADON01015296.1	99	89	AADN03006395.1	87	65	12	*MAGI-1*
Record-134172	ADON01027705.1	99	94	AADN03002000.1	99	79	2	*KIAA1462*
Record-112359	ADON01005944.1	93	93	AADN03002597.1	100	79	2	*ARHGAP21*
Record-106582	ADON01048494.1	100	81	AADN03007512.1	40	65	18	*ACSF2*
Record-111407	ADON01049731.1	51	80	AADN03007334.1	65	72	17	*ASTN2*
Record-88247	ADON01074474.1	97	90	AADN03004436.1	83	78	5	None
Record-135057	ADON01024818.1	99	93	AADN03000109.1	99	82	1	None
Record-130775	ADON01018463.1	100	92	None	None	None	None	None

For Record-134172, 1,964 bp length of flanking sequence was obtained by IPCR. The DNA sequence exhibits 94% and 79% identities with duck and chicken sequences, respectively. *KIAA1462* gene was identified base on the sequence of Record-134172.

For Record-112359, 2,164 bp length of flanking sequence was obtained by IPCR. The DNA sequence exhibits 93% and 79% identities with duck and chicken sequences, respectively. *Rho GTPase activating protein 21*(*ARHGAP21*) gene was identified base on the sequence of Record-112359.

For Record-106582, 2,623 bplength of flanking sequence was obtained by IPCR. The DNA sequence exhibits 81% and 65% identities with duck and chicken sequences, respectively.*Acyl-CoA synthetase family member 2*(*ACSF2*) gene was identified base on the sequence of Record-106582.

For Record-111407, 1,508 bp length of flanking sequence was obtained by IPCR. The DNA sequence exhibits 80% and 72% identities with duck and chicken sequences, respectively. *Astrotactin 2* (*ASTN2*) gene was identified base on the sequence of Record-111407.

The derived sequences of Record-106975, Record-134172, Record-112359, Record-106582 and Record-111407 were deposited in GenBank (http://www.ncbi.nlm.nih.gov/genbank) with the accession numbers KP271033, KP271035, KP271036, KP271032 and KP271034, respectively.

For Record-88247, Record-135057 and Record-130775, 3,100 bp, 1,300 bp and 4,711 bp of flanking sequences were obtained by IPCR, respectively. Three DNA sequences exhibits 90%, 93% and 92% identities with duck sequences, respectively. The DNA sequences of Record-88247 and Record-135057 exhibit 78% and 82% identities with chicken sequences, respectively. We didnotfind the orthologous sequence from the whole-genome shotgun contigs database of chicken (taxid: 9031) for the Record-130775.

## Discussion

### Pool-Based RAD Sequencing

In the present study, we adopted an economical and efficient method of pooled comparative RAD sequencing to discover SNPrelated to goose laying performance. Many studies of mining SNP in animalswithout reference genomes havebeen reported [16,26,27]. Since there is no reference genome available for geese, RAD sequencing provides a cost-effective approach to discover very large number of SNP across goose genome.

The ratio of transition/transversion (ts/tv) is the general property of DNA-sequence evolution[28]. For all the genomic sequences that had been investigated, transitions had been noted to occur at higher frequencies than transversions[10,29,30], because transition did not require a change in conformation[28,31]. In this study, ts/tv ratiois 1.10 for geese, which isin accordance with the rule of transition bias.

The shallow sequencing depth of next-generation sequencing is a major determinant of the quality of genotype calls generated from sequence data [32,33] and the cost of sequencing[34]. Catchen et al. (2011) simulated the RAD-seq process in order totest the ability of USTACKS to identify loci in *threespine stickleback*. They demonstrated thatthe mean sequencing depth of 20× and 40× were dependable for next generation sequencing at low error rate [19]. In this study, the average sequencing depth ofLEBV andHEBV group are20× and 17×respectively, suggesting the reliable sequencing result was obtained upon the depth.

### Discovering Laying-Related SNP

A two-step strategy was applied to discover laying-related SNP by combining pool-based RAD sequencing with individual-based verification in larger population. Next generation sequencing (NGS) of pools of individuals is often more effective in SNP discovery on a genome-wide scale and provides more accurate allele frequency estimates, even when taking sequencing errors into account [33,35,36]. The more cost-effective method of NGS of DNA pools was widely used in various researches[18], which proved that NGS of DNA pools allows estimating the allele frequencies at single SNP with acceptable accuracy, but considerable lower library construction and sequencing efforts. In our study, we identified 467 putative SNP associated with egg production by comparing estimated allele frequencies between LEBV and HEBV DNA pools. 55 out of 467 SNP which were suitable for AS PCR were subjected to individual genotyping in LEBV andHEBV cohorts. 10 SNP exhibited different allelic distribution between the two cohorts, with 18.2% positive rate. Compared with the previous studies, Turner et al. (2010) detected 8.4 million polymorphisms between two DNA pools of *Arabidopsis lyrata* from serpentine and nonserpentine soils. Of the 8.4 million polymorphisms, 96 have allele frequency differences of greater than 80% between soil types. At the same time, 81 genes were found based on 96 polymorphisms [37]. There are no uniform standards can be obtained to evaluate the numbers of positive rate polymorphisms from pool-based sequencing. However, Gautier et al. (2013) assessed the accuracy of allele frequency estimation based on the data of pool and individual-based RAD sequencing. The results showed that DNA pool sequencing is an economical and effective method to estimate allele frequencies for massive number of SNP sites[38]. Zhu et al. (2012) experimentally identified that DNA pool sequencing is a very powerful and cost-effective technique for SNP discovery on genome-wide scales [39].

In this study, 10 candidate SNP identified by comparative study with small-scale representative individuals were further verified in a larger goose population with egg production. 8 SNP showed significant effect on egg numbers with a positive rate of 80%, which resulted in a high concordance between small-scale representative comparison and large-scale verification. We conclude that pool-based RAD sequencing combined with extreme representative individual comparison is a cost-effective way to identify associate SNP for the traits of interest.

### Association Analysis with Egg Production

To improve laying performance is of great significance for goose production. Nevertheless, due to the low heritability of reproductivity, phenotype-based selection is of low efficiency for improving laying performance. Identification of genetic markers or genes involved is conducive to improvement of such a trait of low heritability. Numerous researchers had been focused on exploring genetic mechanisms of geese reproductive trait. Jiang et al. (2011) detected SNP in the 5'-flanking region of *PRL* gene to find genetic marker influencing on reproduction traits in the *Wan-xi* White goose [40]. Chen et al. (2012) revealed the significant association between SNP in *PRLR* exon 10 and egg performance of *Wanjiang* white goose [3]. Xu et al. (2013) performed *de novo* transcriptome assembly and gene expression analysis and identified a large number of genes associated with follicle development and reproductive biology including *cholesterol side-chain cleavage enzyme* and *dopamine beta-hydroxylas*[9]. Kang et al (2014) demonstrated that *enolase1* (*ENO1*) gene expression was higher than in the ovaries of laying geese compared with prelaying geese, and identified expression profiling of the *ENO1* gene in the ovarian follicle of the Sichuan white goose [41]. In our study, we clearly demonstrated that 8 SNPdisplayed significant effects on laying trait in geese. The linear regression procedure further revealed two multiple-SNP networks for egg number in which Record-111407, 106975 and 112359 were involved. The model prediction showed good agreement with the observed values, which verified the combination effects of these SNP on egg number. Previous studies also reported multiple genes or markers can be used for predication of traits. Jiang et al. (2009) confirmed two-gene or three-gene networks significantly affected 5 or 8 traits in beef cattle through the regression analysis of multiple markers [25]. Ghazalpour et al. (2006) constructed a gene co-expression network in mouse liver with microarray and genetic marker data, and examined the relationship of several gene modules and body weight of mouse[42]. Therefore, these 8 SNP, especially the combination of Record-111407, 106975 and 112359, could be promising molecular markers for the selection of goose laying performance. We further explored the functional association of *MAGI-1*, *ARHGAP21* and *ASTN2* derived from Record-111407, 106975 and 112359 respectively, by using DAVID Bioinformatics Resources 6.7 [43] and UniHI online tool [44].The analysis resultsshows these genes are not directlyassociated in any signal pathway or gene network.However, *MAGI-1 and ARHGAP21* can be directly or indirectly regulated by *Stratifin* (*SFN*) gene. It has been reported that the expression of *SFN* is frequently lost in various types of human diseases including ovarian cancer[45], uterine papillary serous carcinoma[46], uterine leiomyomas [47], ovarian granulosa cell tumors and steroid cell tumors[48]. Wang et al. (2012) indicated the expression of *SFN* was negatively correlated with *estrogen* and *progesterone receptor* (*ER* and *PR*)[47]. Khongmanee et al. (2013) revealed that *SFN* play an important role in anoikis resistance of cholangiocarcinoma cells[49]. A lot of evidences show the strong possibility that*MAGI-1* and *ARHGAP21*will play a role in disease and function of reproduction.

### Gene Cloning

A total of 5 novel genes were obtained for geese using IPCR extension of RAD tags combined with comparative alignment of public database of ducks (taxid: 8835) and chicken (taxid: 9031). Compared with the previous studies, we did not detect the well-defined reproductive-related genes like *FSHβ*[4], *PRL* [4], *GnRH* [50], *LH*[51] and *PRLR*[51]. Instead, we found three novel genes, *MAGI-1*, *ARHGAP21* and *KIAA1462*, may play important roles in egg production.Actually, RAD-sequencing is a methodthat creates a reduced representation of genome by restriction enzyme digestion.The SNP obtained by this method only represent a small portion of the whole genome. In this study, 884,827 and 942,117 RAD-tags were obtained from the LEBV and HEBV DNA pool, respectively. The average coverage rate was estimated to be ~6.96% of the whole genome(1.1Gb, *Anas platyrhynchos*[52]).Therefore, it is of high possibility that the previouswell-known laying related genes could not be included in the gene list obtained.

For the *MAGI-1*gene, Kranjec et al. (2014) demonstrated itcan promote the cell-cell contact in HPV-positive cells, thereby has the function of represseing cell proliferation and promoting apoptosis [53].*ARHGAP21*functions preferentially as a *GTPase-activating protein* (*GAP*) for *CDC42* and regulates the *ARP2/3* complex. It is localized in the nucleus, cytoplasm, or perinuclear region and participates in cell-cell adhesion formation and cellular migration[54,55]. *KIAA1462* is a protein-coding gene which is localized in the nucleus, cytosol and plasma membrane. Diseases associated with *KIAA1462* include artery disease and coronary artery disease [56–58]. Akashi et al. (2011) identified the *KIAA1462* as a novel protein localized at cell–cell junctions, and concluded that the accumulation of *KIAA1462*into endothelial cell–cell junctions depends on VE-cadherin-mediated cell–cell adhesion [59]. Oocyte growth is supported by theca cells and granulosa cells, which established dynamic and highly organized cell layers surrounding the oocyte.Gap junctions between oocytes and granulosa cells is complex, and plays a major role in the support of oocyte growth, the maintenance of meiotic arrest, and signal transduction throughout the follicular epithelium[60,61]. A well-known effect associated with the establishment of cell-cell junction is the inhibition of cell proliferation [53,62,63]. These above evidences show that these genes (*MAGI-1*, *ARHGAP21* and *KIAA1462*) have a high possibility to affect the granulosa cell proliferation and apoptosis, then interfere with oocyte growth. In addition, *KIAA1462* plays a very important role in meiotic recombination. Chowdhury et al. (2009) found*KIAA1462* was one of six loci associated with variation in human recombination rates [64]. Failures or errors in meiosis can lead to infertility, miscarriages, or birth defects [65,66].

Theother two cloned genesinclude*ACSF2* and *ASTN2*.*ACSF2*is the member of Acyl-CoA synthetases (*ACS*) family, which is involved in fatty acid synthesis and the tricarboxylic acid cycle [67]. *ACSF2* is a mitochondrial matrix enzyme and located inthe mitochondrial matrix [68]. The characteristic of *ACSF2* found in the engery metabolism processes, and be related with mitochondrial function, suggested this gene may play a role in the reproduction. *ASTN2* is expressed at high levels in migrating, cerebellar granule neurons [69]. It plays an important role in neuronal functioning [70,71]. Lesch et al. (2008) identified the *ASTN2* gene participates in cell adhesion and neuronal cell–cell communication [72]. Ahn et al. (2010) found a novel microRNA that was derived from an intron within *ASTN2* gene, and was preferentially expressed in the gonads [73].

## Conclusions

We applied the pool-based RAD sequencing strategy for SNP discovery in geese. Eight laying-related SNP were verified by individual-based association analysis. Five novel genes for geese were cloned based on the laying-ralated SNP. Our data suggested that these SNP or genes might be promising candidate markers or targets for marker-assisted selection of animals prolific in production of egg numbers in geese.These methods could be performed in other production animals to help identify more efficient, greater performing animals for human consumption/use. Our studies also demonstrate that molecular methods can serve useful purposes for reasons (other than) simply determine molecular mechanisms underlying some physiological cascade. Indeed, more research in this vein will help in the production of all sorts of animals.

## Supporting Information

S1 Fig Agarose gel electrophoresis of IPCR products in the eight SNPS. M1 indicates 1kb DNA maker. M2 indicates 250bp-I DNA ladder maker.
(TIF)

S1 Table SNPs and their primers information for the geese. (XLSX)

S2 Table Primers of inverse PCR. (XLSX)
